# Supplementary material for: Structural biases in disordered proteins are prevalent in the cell
Source: Nat Struct Mol Biol. 2024 Jan 4;31(2):283–92. doi: 10.1038/s41594-023-01148-8 (PMC10873198; doi:10.1038/s41594-023-01148-8)
Supplement: Supplementary file 1 — Supplementary Figs. 1–23 and Table 5. [file 41594_2023_1148_MOESM1_ESM.pdf]

nature structural & molecular biology

Article

https://doi.org/10.1038/s41594-023-01148-8

Structural biases in disordered proteins are prevalent in the cell

In the format provided by the authors and unedited

Supplementary Information for

Structural biases in disordered proteins are prevalent in the cell

David Moses[1,2,*], Karina Guadalupe[1,2,*], Feng Yu[2,3], Eduardo Flores[1,2], Anthony Perez[1,2], Ralph McAnelly[1], Nora M. Shamoon[2,4], Gagandeep Kaur[1], Estefania Cuevas-Zepeda[1], Andrea D. Merg[1,2], Erik W. Martin[5,†], Alex S. Holehouse[6,7], Shahar Sukenik[1,2,3,8,✉]

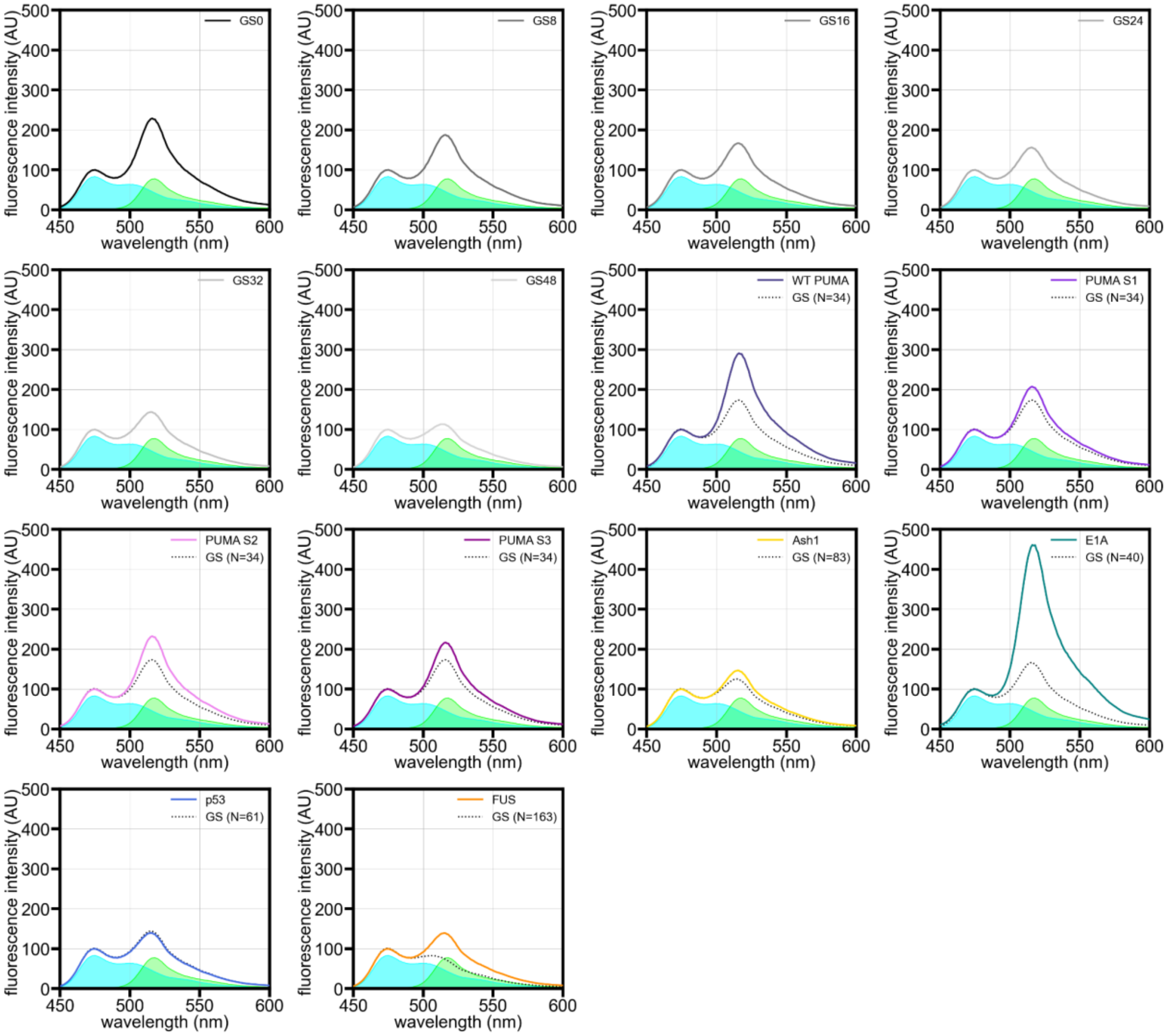

Figure S1. Fluorescence spectra of FRET constructs *in vitro*. Spectra of constructs incorporating IDRs are compared with interpolated fluorescence spectra of FRET constructs incorporating GS-repeat sequences of equal length (black dotted curves), where N refers to the number of amino acids. Blue and green shaded areas are the base spectra for mTurquoise2 and mNeonGreen, respectively, in the same buffer solution.

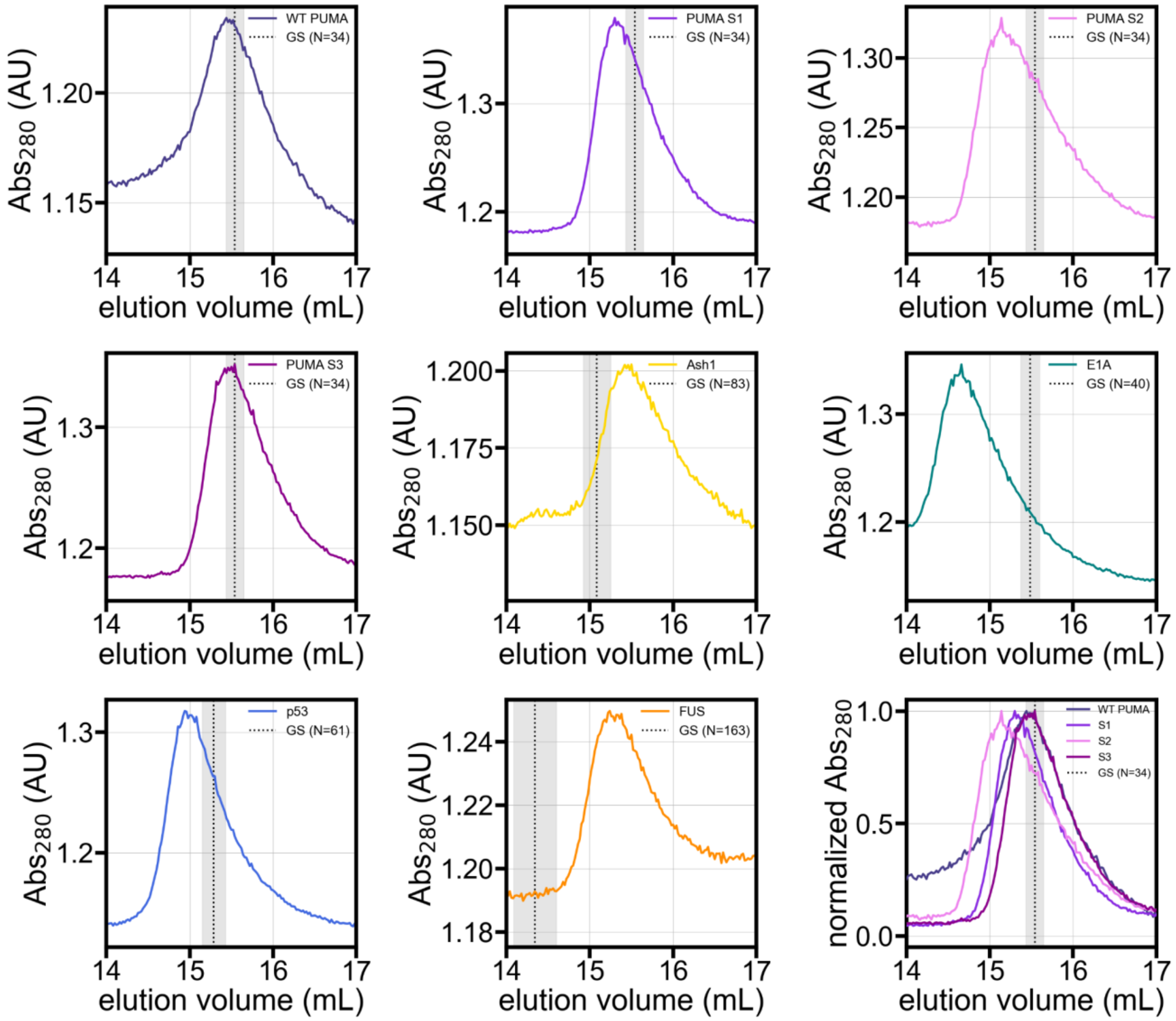

Figure S2. Chromatograms from SEC-SAXS experiments in which the samples were donor-IDP-acceptor FRET constructs in a dilute phosphate buffer solution. Vertical dotted line labeled "GS" in each panel represents the expected elution peak position of a FRET construct containing a GS-repeat sequence equal in length to the IDP, where N refers to the number of amino acids. Shaded region in each panel represents the standard error of the expected GS peak position. The bottom right panel shows all PUMA constructs normalized to their minimum and maximum value.

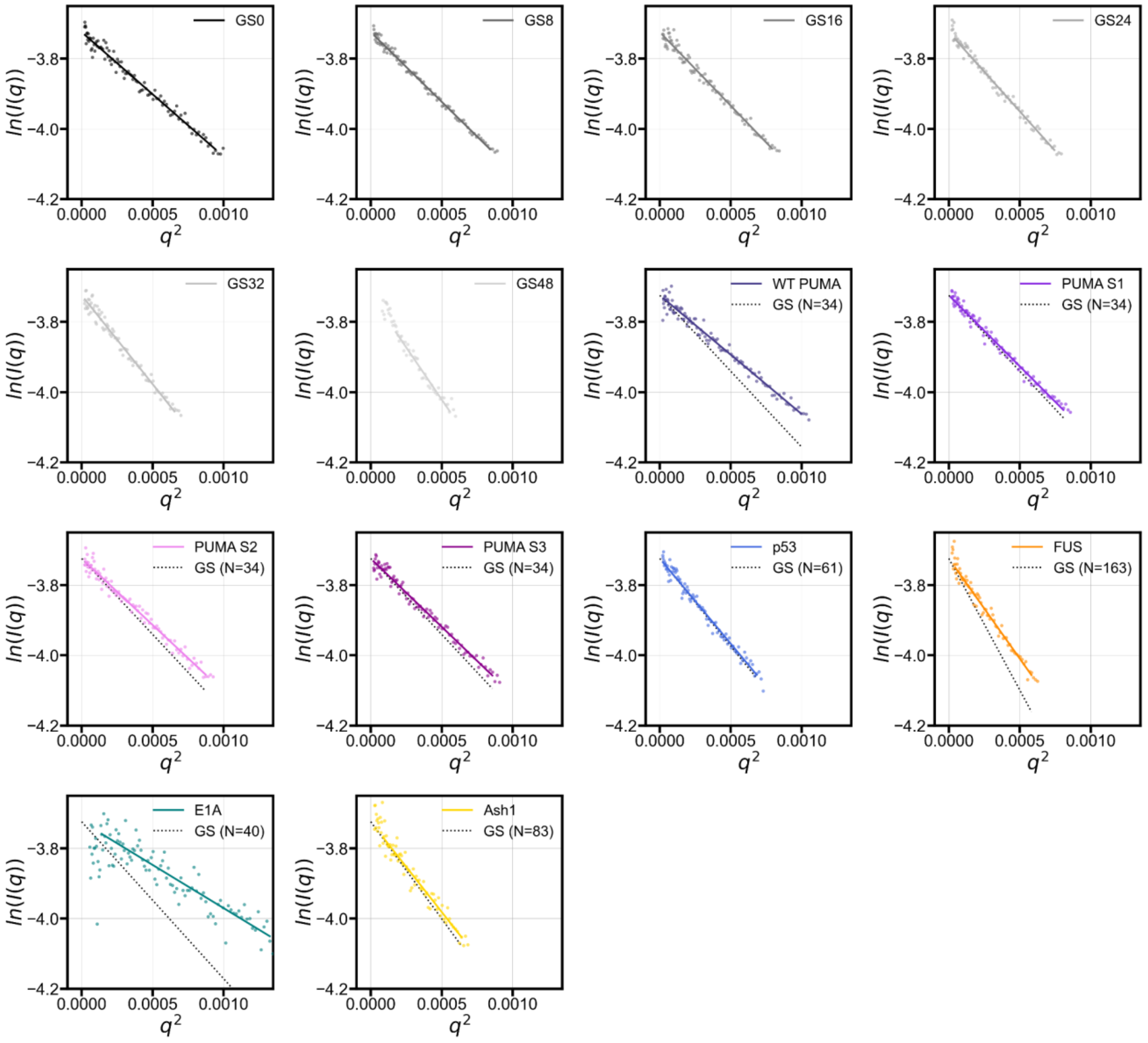

Figure S3. Guinier plots for donor-IDP-acceptor FRET constructs from SEC-SAXS experiments. Points are obtained from scattering data averages, and lines are linear fits of the data to a $q * R_g$ value of 1. For IDRs that are not GS-repeat sequences, a black dotted line denotes the extrapolated fitted line for a GS-repeat sequence of the same length.

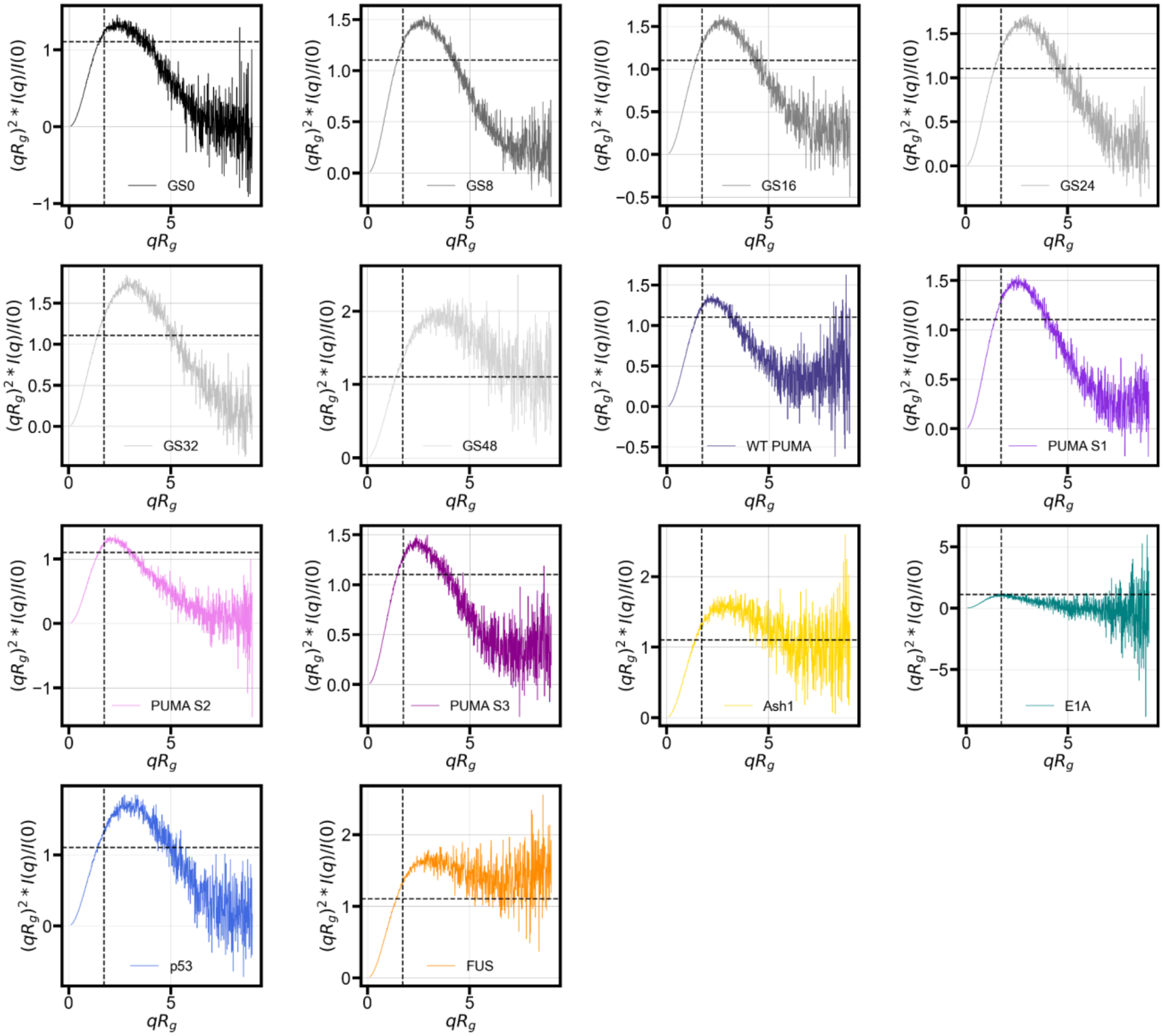

Figure S4. Dimensionless Kratky plots derived by transforming the scattering profiles from which the R_g values reported in the main text were calculated. For a globular protein, the peak position should be at $qR_g = \sqrt{3} \sim 1.73$ (shown by vertical dashed line) and the peak height should be $(qR_g)^2 * I(q)/I(0) = 3/e \sim 1.1$ (shown by horizontal dashed line).

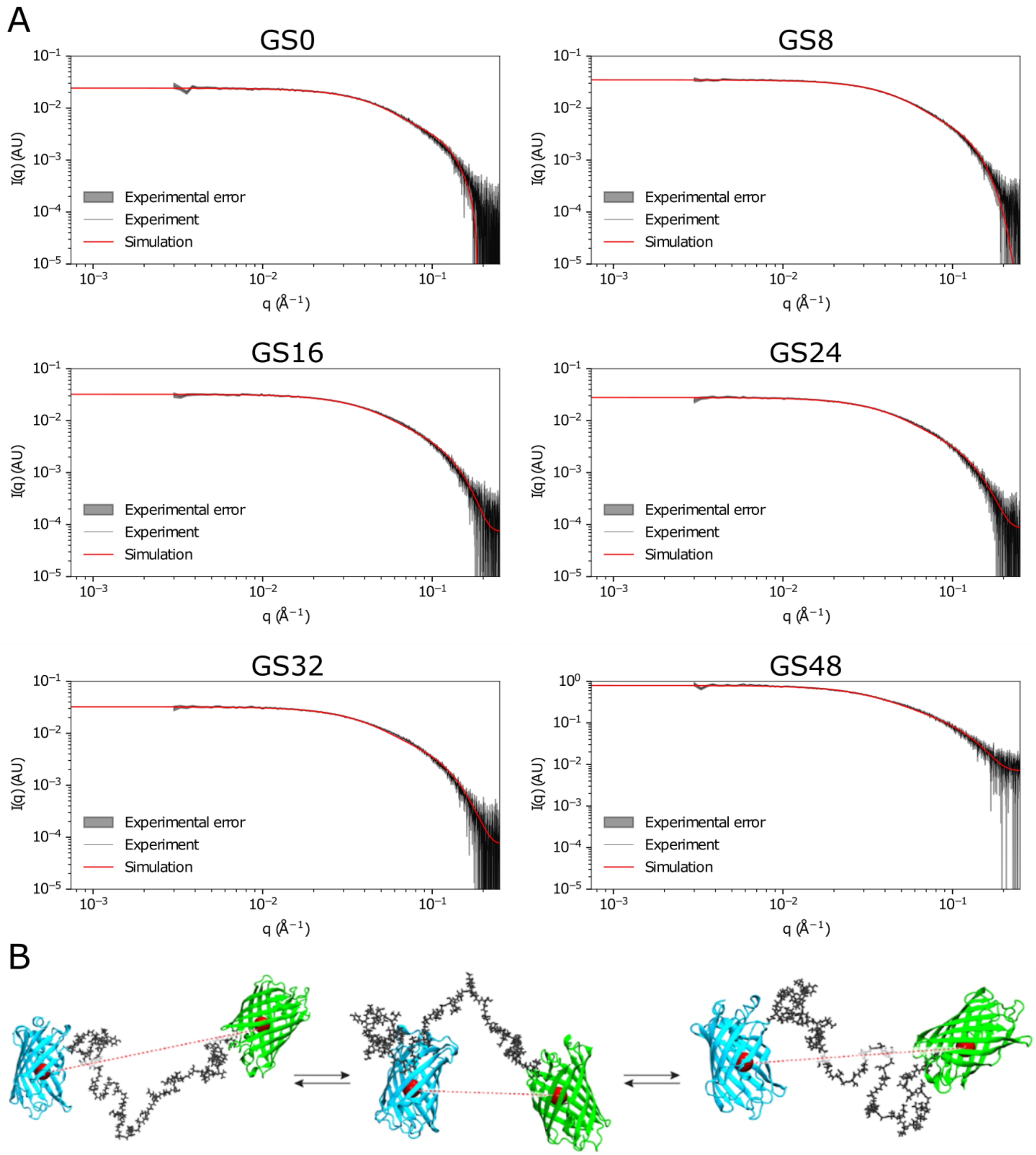

Figure S5. (**A**) Comparison of experimentally measured small-angle X-ray scattering profiles (black lines with shaded regions representing measurement error) and simulation-derived sub-ensembles (red lines) for GS-repeat sequences of different lengths. (**B**) Example snapshots from simulations. R_e^{app} is calculated based on the distance between residues from the center of the two beta-barrels (dashed red line).

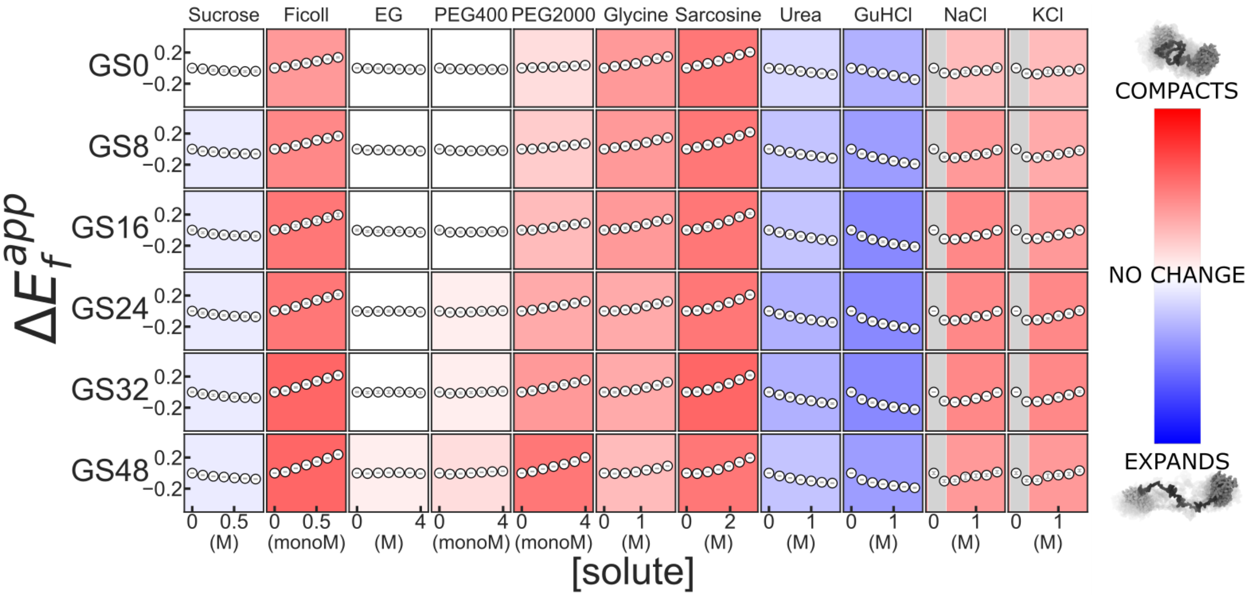

Figure S6. Solution space scans of GS-repeat homopolymers. We measured the FRET signal of IDP constructs in the presence of denaturing (urea, guanidinium), stabilizing (sucrose, ethylene glycol, glycine, sarcosine), and crowding (PEG400, PEG2k, Ficoll) solutes, as well as salts (NaCl, KCl) that screen electrostatic interactions.Each cell shows $\Delta E_f^{app} = E_{f,solute}^{app} - E_{f,buffer}^{app}$ as a function of increasing solute concentration. Blue/red background indicates expansion/compaction. monoM: Molar concentration of a polymer expressed as a concentration of monomeric units. Light gray shaded regions on left side of cells for solutes NaCl and KCl: approximate range of concentrations within which electrostatic screening is the dominant effect; the leftmost two points of each series, since they are within that range, are not used in the assignment of background color. Error bars indicate the spread of the data over two independent repeats.

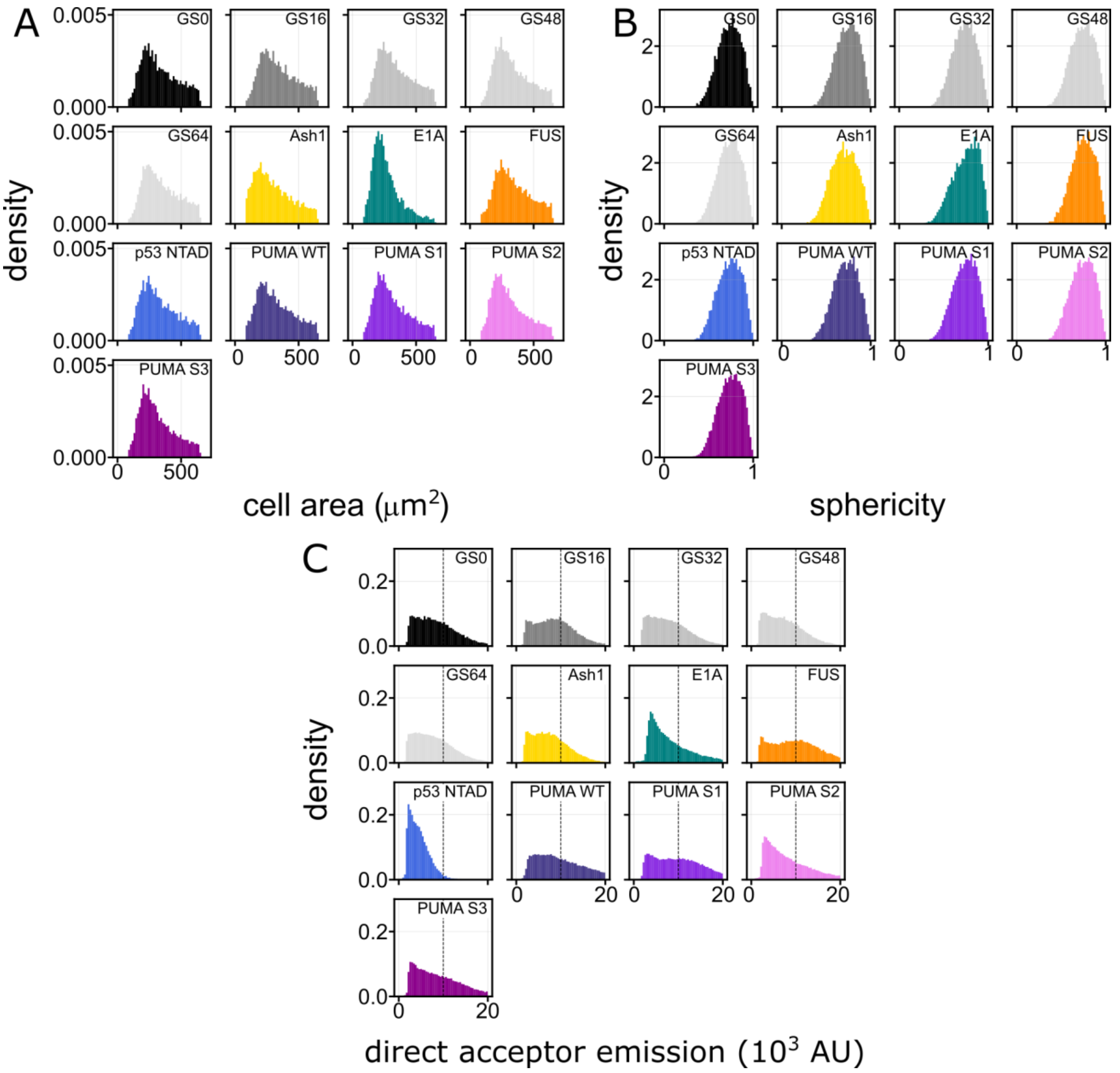

Figure S7. Probability density of cellular features across cells expressing different FRET constructs. Each histogram contains over 10^3 cells (N's are available in Table S4). (**A**) Cell area (in μm^2). (**B**) Cell circularity calculated as $4\pi(area/perimeter^2)$. (**C**) Direct acceptor emission (cells excited at 511 nm) is a metric for in-cell construct concentration. To eliminate artifacts resulting from high overexpression, and to facilitate accurate comparison with *in vitro* measurements done at 1 uM, we left cells with a direct acceptor emission higher than 10,000 out of the analysis, indicated by the dashed vertical line (see also **Fig. S17**).

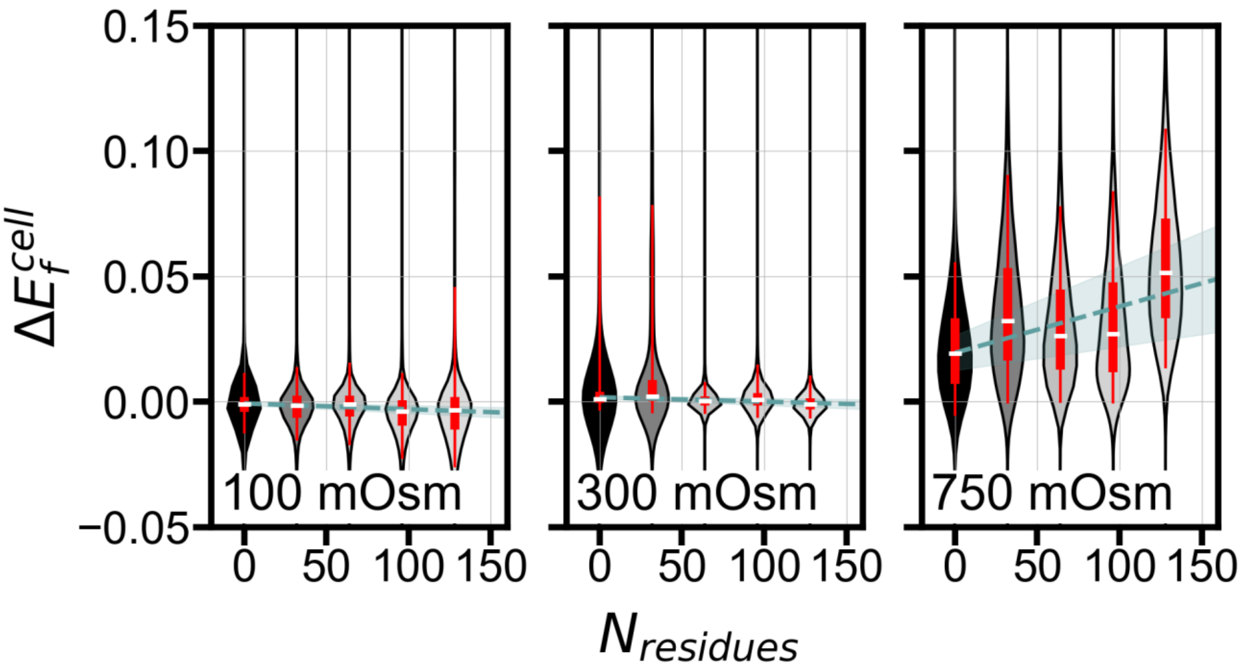

Figure S8. Linear fit of ΔE_f^{cell} as a function of GS linker length for hypo, iso, and hyperosmotic perturbations. Dashed green line is a linear fit of the medians, shown as white dashes, and shaded areas are the errors of the fit. For all violins, the median is shown as a white horizontal line and the thick and thin red bars span median 50% and 95% of the data, respectively. For the number of cells used to generate each violin plot, see **Table S4**.

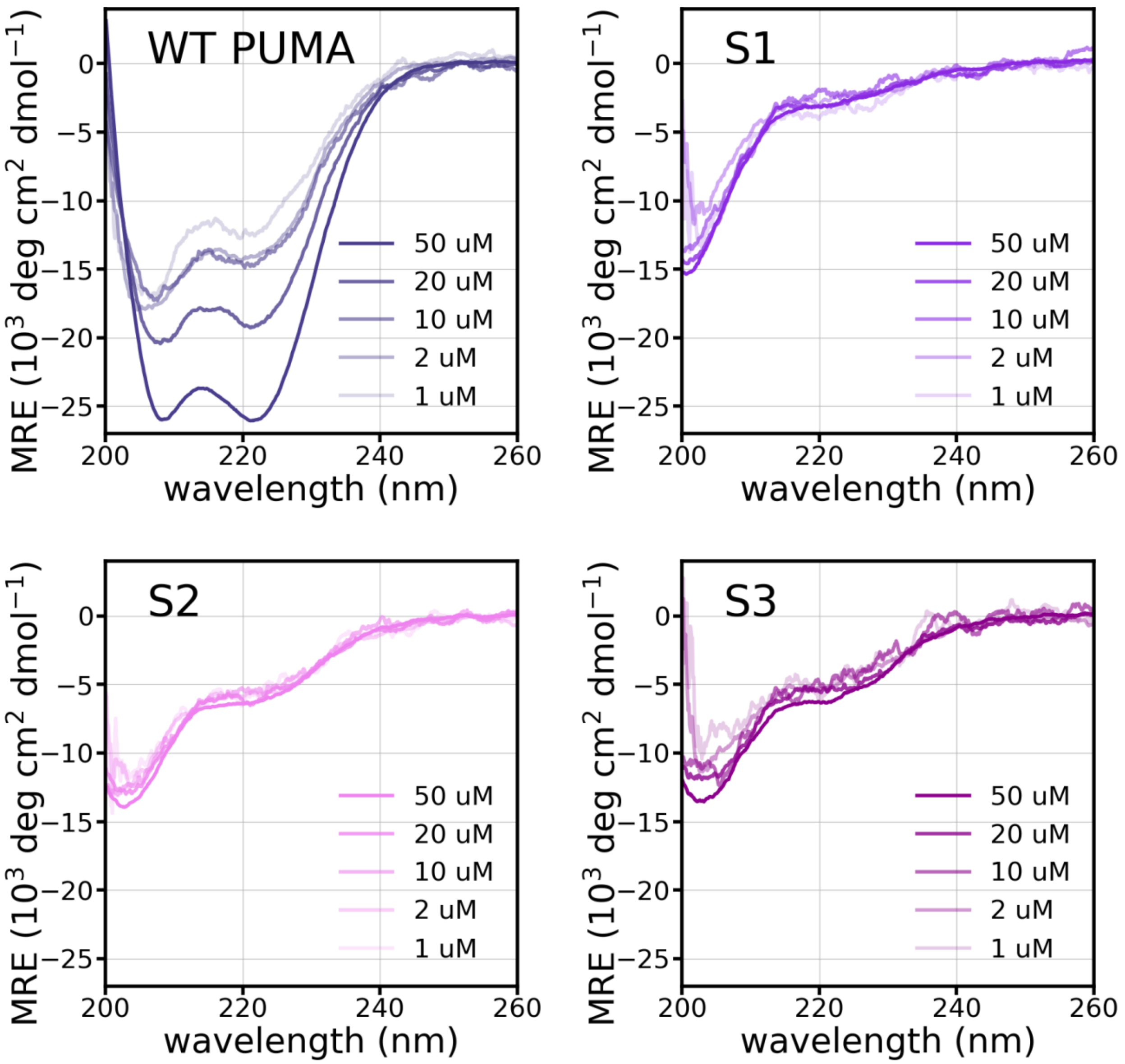

Figure S9. Concentration dependence of circular dichroism measurements of PUMA WT and sequence scrambles. The concentration dependence seen for WT PUMA may be a result of dimer formation at concentrations > 10 μM, which has been previously reported[1]. The WT PUMA construct in all *in vitro* experiments is used at a concentration of 1 μM. At this concentration, no change in CD spectra is seen, and so *in vitro* experiments are expected to have the monomeric construct. All other constructs showed no concentration-dependent change in spectra.

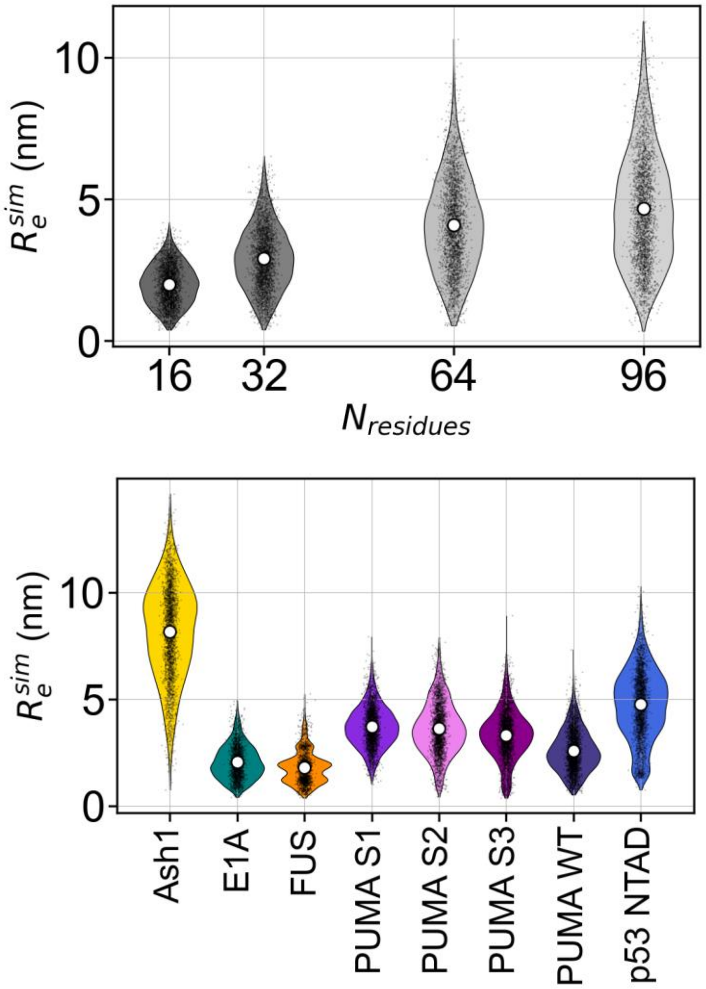

Figure S10. All-atom simulations of label-free IDRs using the ABSINTH forcefield[2]. Violin plots are obtained from random sampling of 2000 frames from ensembles containing at least 20,000 conformations, shown as the overlayed black points. White circles represent the mean of the data. FUS shows a multimodal, highly compact distribution since its ensemble is collapsed and poorly sampled and is likely not indicative of the ensemble for this construct.

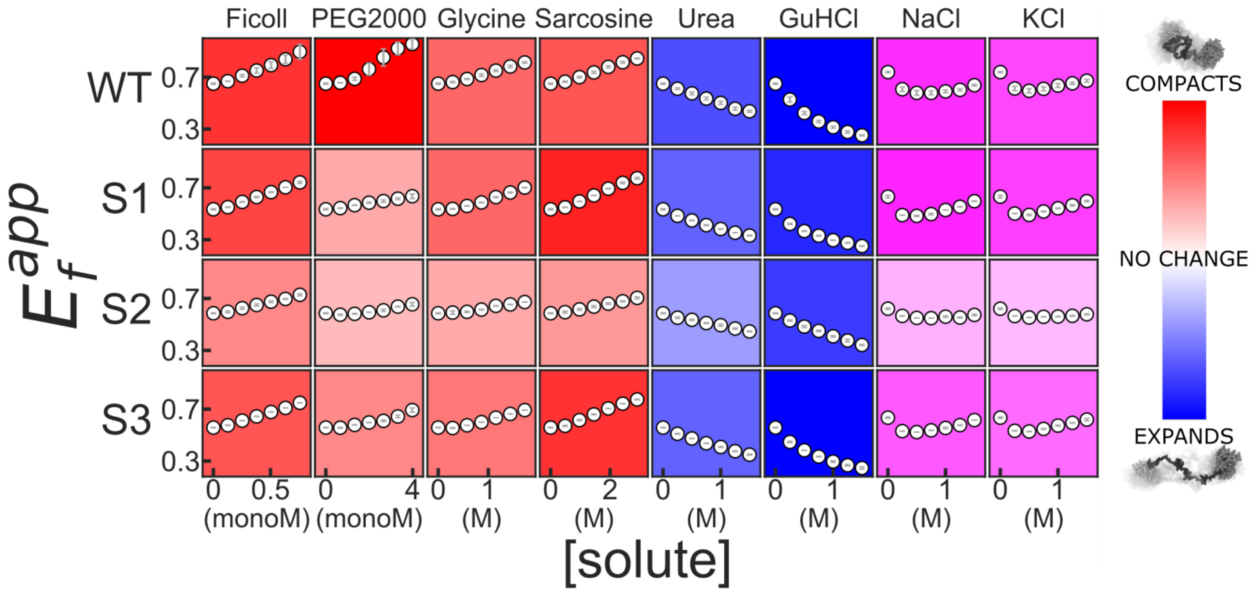

Figure S11. Solution space scans of WT PUMA and sequence scrambles. Each cell shows E_f^{app} as a function of increasing solute concentration. Blue background indicates expansion and red indicates compaction, with deeper shades indicating more change. Purple background indicates a non-monotonic response, with deeper shades representing more curvature. monoM: Concentration of a polymer expressed as a concentration of monomeric units. Grey error bars indicate the spread of the data over two independent repeats.

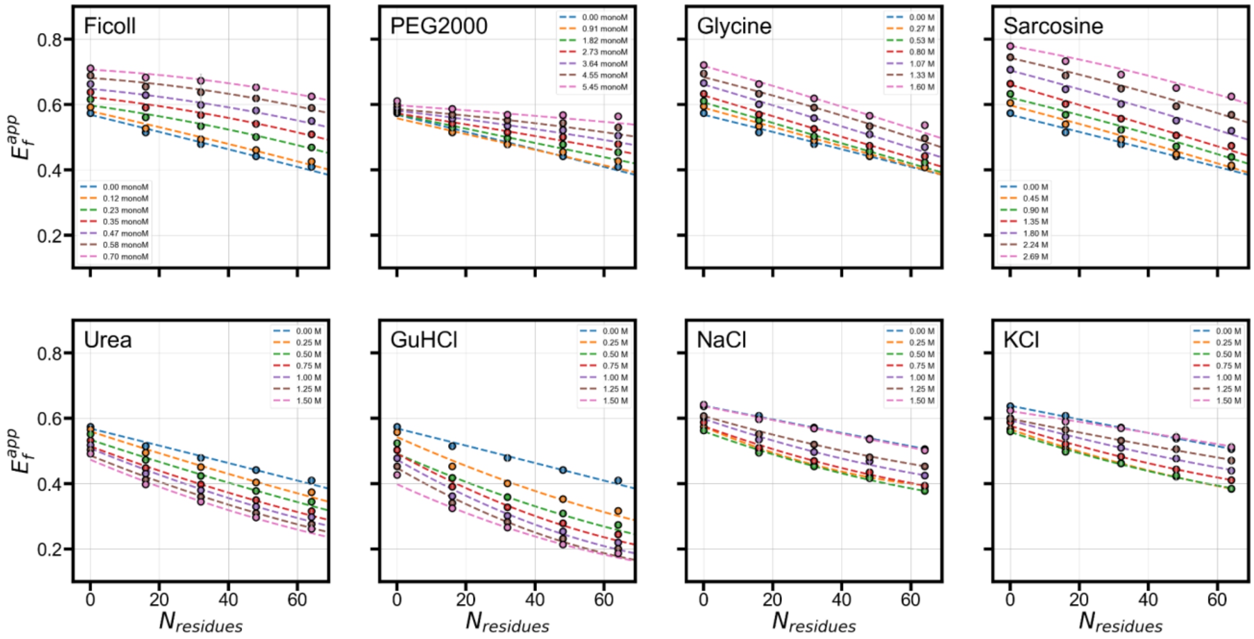

Figure S12. E_f^{app} vs. length of GS-repeat sequence in various solution conditions. A second degree polynomial fit, shown as dashed lines, is used for interpolation of ΔE_f^{app} for arbitrary sequence lengths in **Figs. 3H** and **4F**.

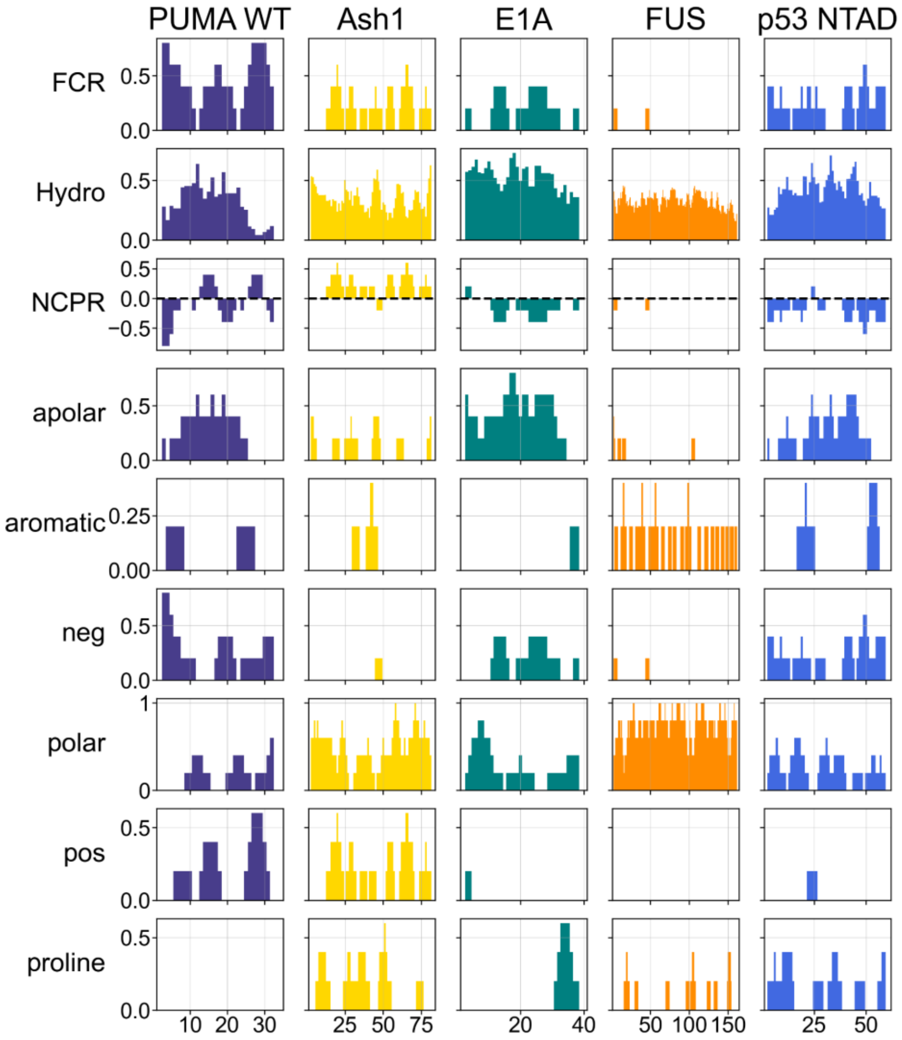

Figure S13. Sequence features of IDP sequences in **Fig. 4**, calculated using locaCIDER[3]. All bars represent the average over a five-residue window centered at the specified residue number. FCR: Fraction of charged residues; Hydro: Kyte-Doolitle hydrophobicity scale; NCPR: net charge per residue; apolar: fraction of ALMIV residues; aromatic: fraction of FYW residues; neg: fraction of ED residues; pos: fraction of KR residues; polar: clusters of QNSTGHC residues; proline: fraction of P residues.

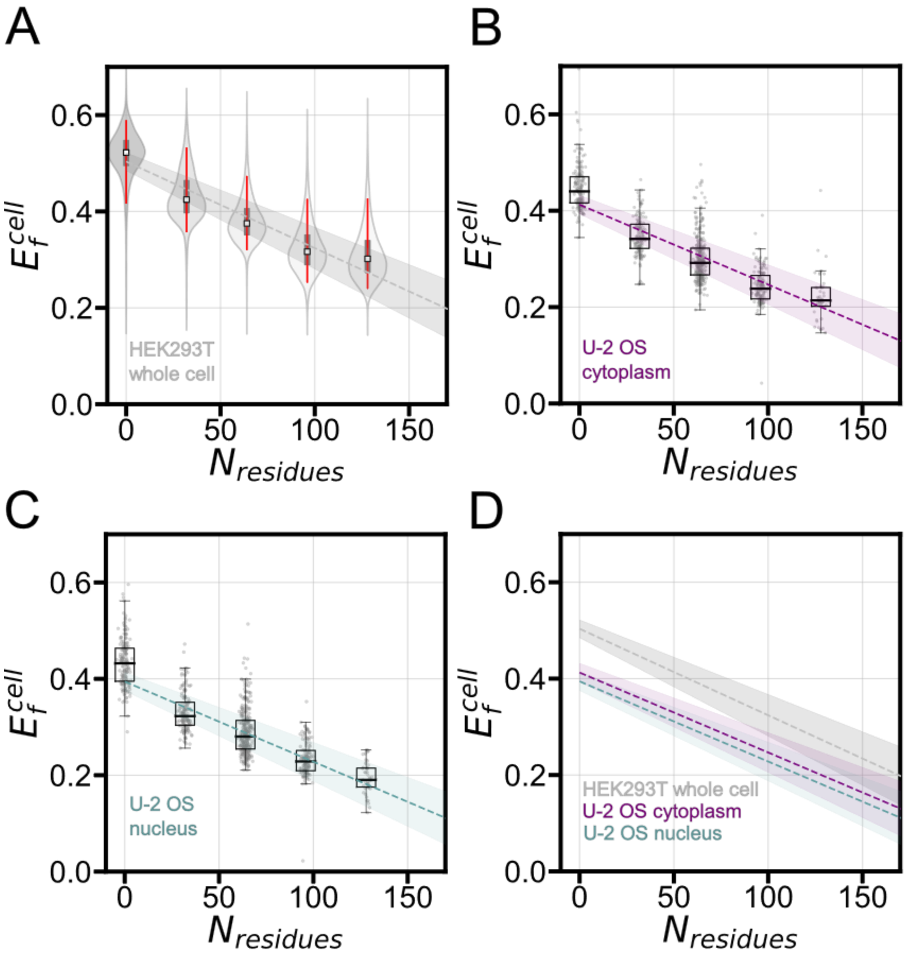

Figure S14. Comparison of E_f^{cell} from GS-repeats in HEK293T and U-2 OS cells. (**A**) HEK293T live cell measurements taken at 10x magnification. All data and features of violins are identical to **Fig. 2G**. The gray line is a linear fit of the medians, and fit error is shown by the shaded region. (**B-C**) Cytoplasm and nucleus measurements taken in U-2 OS cells at 40x magnification. The box spans the median 50% of the data, the whiskers span the minimum and maximum, and the black line at the center shows the median. The purple and blue lines are the linear fits of the medians and fit error is shown by the shaded regions. Points correspond to individual cells. Images were taken at 40X with N > 25 for each box plot. (**D**) The linear fits of the medians with fit error shown by the shaded regions for HEK293T (gray), U-2 OS cytoplasm (purple), and U-2 OS nucleus (blue). The data used to generate the nucleus and cytoplasm box plots is in source data for **Fig. 4.** N's for each violin and box plot is in **Table S4.**

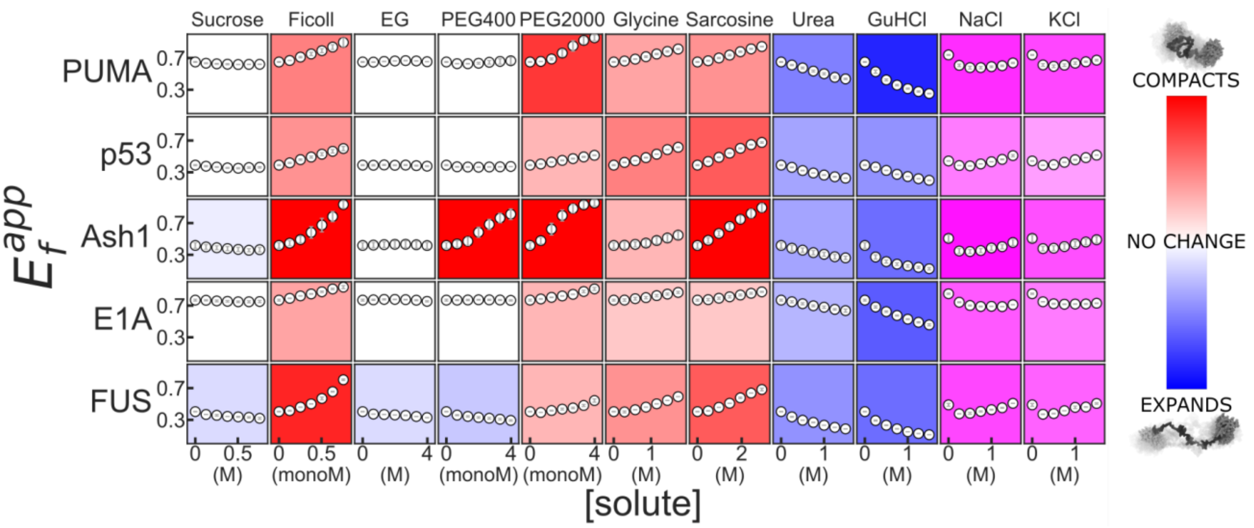

Figure S15. Solution space scans of naturally occurring IDRs. Features are as in **Fig. S11**.

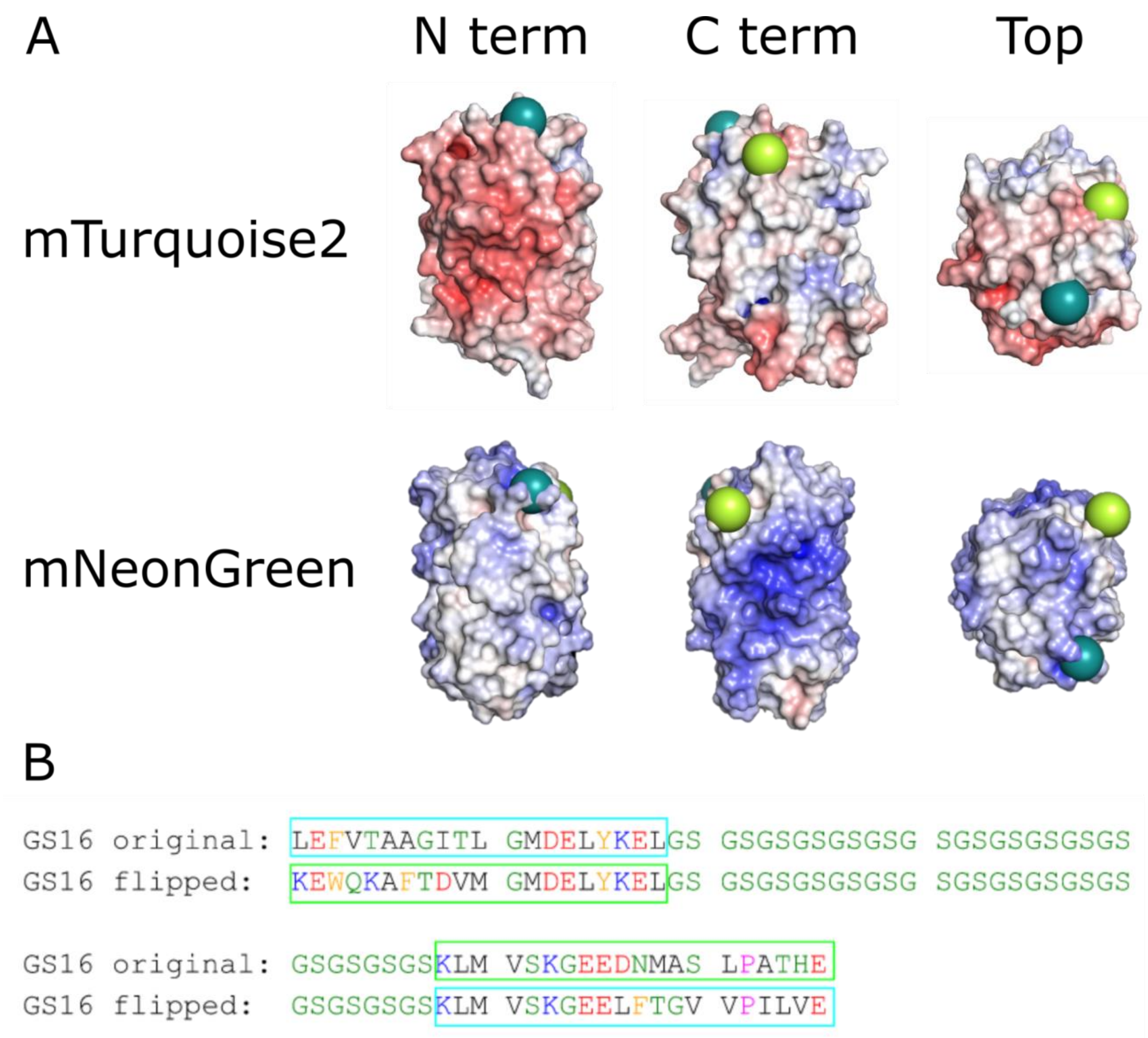

Figure S16. Original and flipped GS16 repeat constructs. (**A**) Surface electrostatic analysis of PDB files for mTurqouise2 (4AR7)[4] and mNeongreen (5LTR)[5] using APBS[6] shows different surface charges for mNeonGreen and mTurquoise2. The N and C termini are labeled as yellow and cyan spheres, respectively. (**B**) Sequences of GS16 with the nearest 20 residues from the flanking fluorescent proteins, aligned using Clustal Omega[7]. Color codes are from CIDER analysis[3]. Red: negative charge; blue: positive charge; black: hydrophobic residues; green: polar residues; orange: aromatic residues. Cyan and green boxes show residues at the terminals of mTurquoise2 and mNeonGreen, respectively, connected to the GS-repeat sequence.

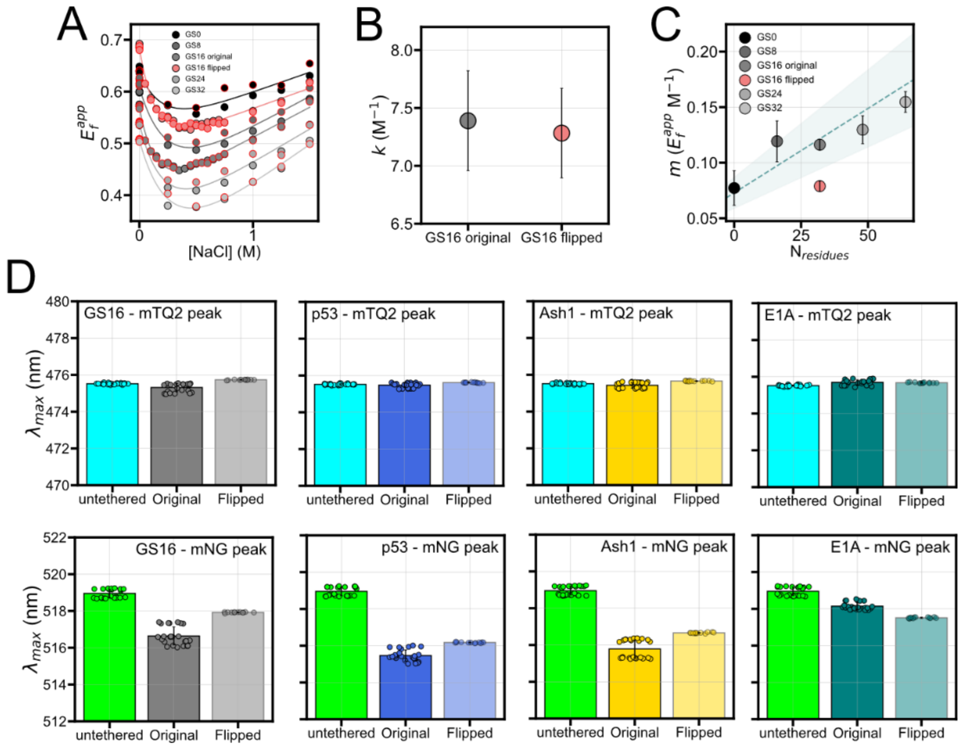

Figure S17. (**A**) FRET efficiencies of GS-repeat constructs as function of NaCl concentration. Experimental data shown as symbols was fit to an exponential decay with a sloping baseline, $E_f^{app}([NaCl]) = Ae^{(-k[NaCl])} + m[NaCl] + b$. In this equation, used previously to describe salt-dependence behavior of protein structure, k is a decay constant that indicates the effect of screening of electrostatic interactions on ensemble structure, and m is a linear slope that accounts for the specific interactions of the ions at higher concentrations[8–10]. Different edge colors show results of two independent repeats. (**B**) Comparison of the decay constant k of the original and flipped GS16 constructs. Value and errors are calculated from a global fit of two independent repeats. Identical k for the original (7.4 ± 0.4 M^{-1}) and the flipped construct (7.3 ± 0.4 M^{-1}) indicates that electrostatic interactions cannot explain the difference in E_f^{app} between the two constructs (**C**) Slope m vs. the length of all GS-repeat sequences. All original GS repeats show a linear relationship between m and length. The flipped GS16 construct falls below this line, indicating a tighter packing of one or both of the FPs. (**D**) Comparison of the peak emission wavelengths for mTurquoise2 (top) and mNeonGreen (bottom) untethered vs. in the original and flipped constructs compared in **Fig. 5B**. For mNeonGreen, P < 0.0001 for untethered vs. each original construct and untethered vs. each flipped construct (n=24 for mNeonGreen untethered, n=24 for each original construct, and n=12 for each flipped construct). The shifts of the mNeonGreen peak indicate changes in mNeonGreen between the original and flipped construct as a result of IDP presence. Error bars represent the standard deviation of the repeats for each construct.

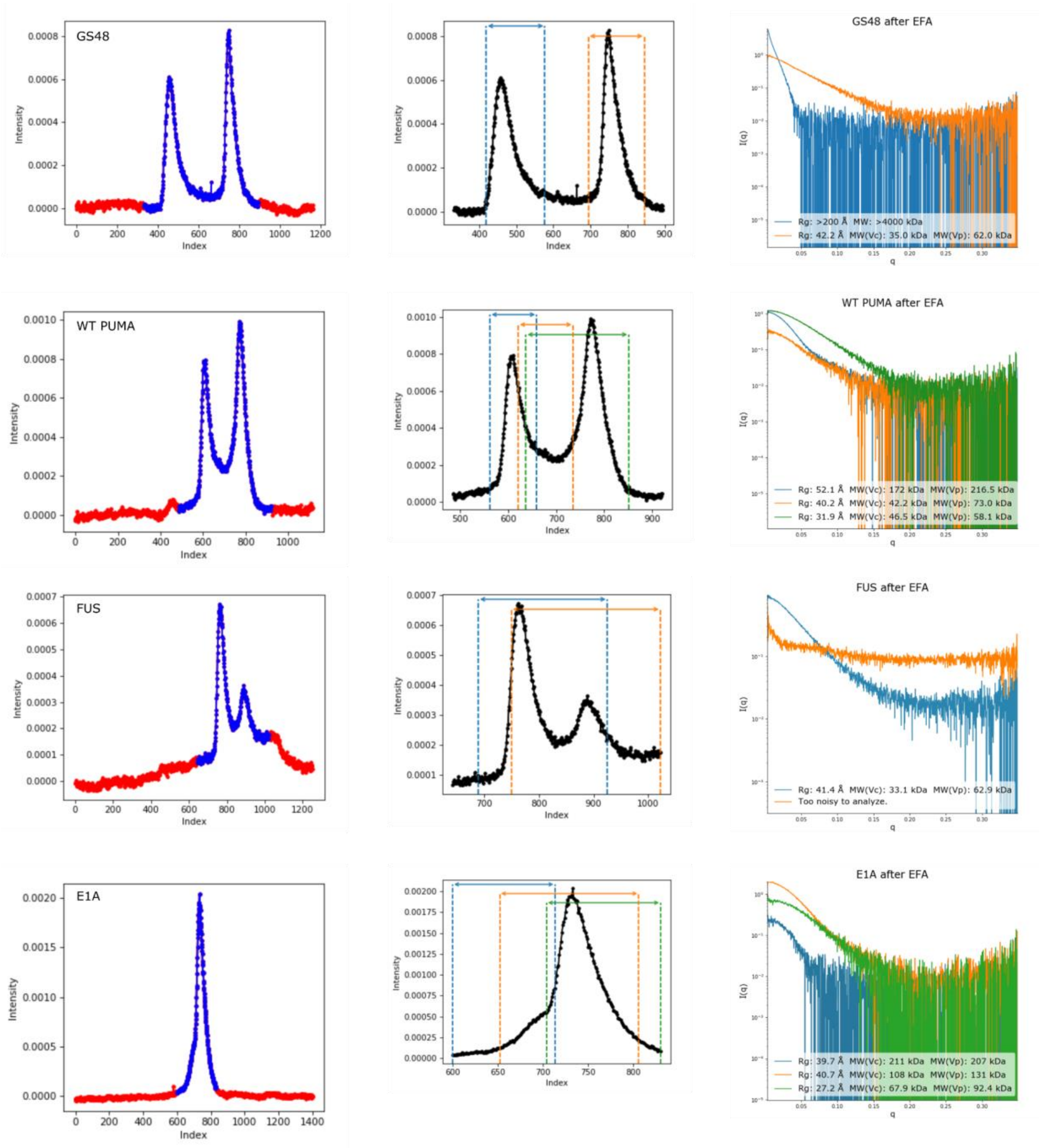

Figure S18. Screens from BioXTAS RAW software[11] showing process of deconvolution of SEC peaks using evolving factor analysis. Left: raw chromatograms. Center: ranges of deconvoluted peaks. Right: $I(q)$ *vs.* q series, calculated radius of gyration, and calculated molecular weight for each deconvoluted peak. Same colors in center and right panels represent the same deconvoluted peaks.

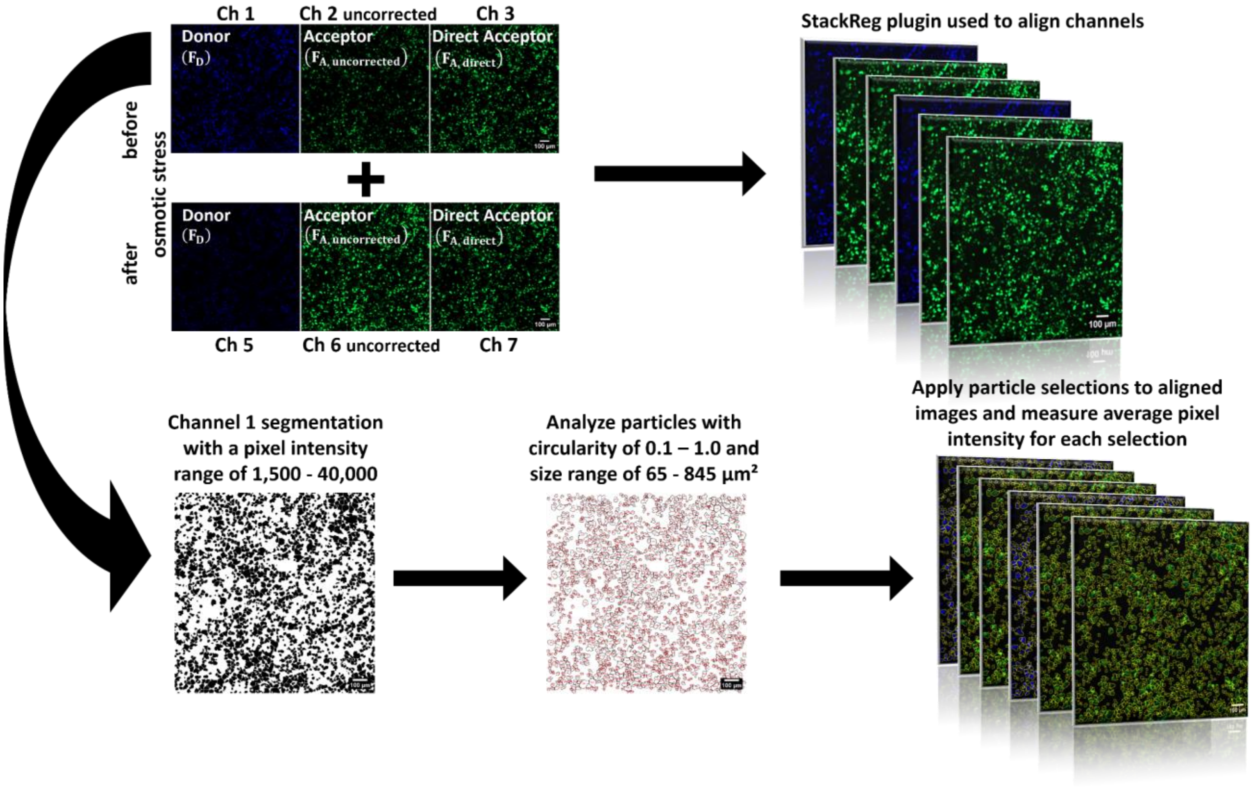

Figure S19. Analysis pipeline for live cell data. The donor channel before perturbation (Ch 1) was segmented using a fixed threshold to include any pixels with an intensity value between 1,500 - 40,000. The ImageJ "analyze particles" algorithm was used to select thresholded regions with a circularity between 0.1 -1.0 and a size of 65 - 845 µm². All channels were aligned using the StackReg plugin before segmented regions were applied and measured. Final measurements were corrected for bleedthrough and cross-excitation using slopes obtained from **Fig. S19.** The complete dataset can be found in **Table S3**. In this table, channels 1, 2, 2 uncorrected and 3 correspond to donor (F_D), corrected acceptor ($F_{A,}$), uncorrected acceptor ($F_{A,uncorrected}$) and direct acceptor ($F_{A,direct}$) before osmotic stress, respectively. Channels 5, 6, 6 uncorrected and 7 correspond to donor (F_D), corrected acceptor ($F_{A,}$), uncorrected acceptor ($F_{A,uncorrected}$) and direct acceptor ($F_{A,direct}$) after osmotic stress, respectively.

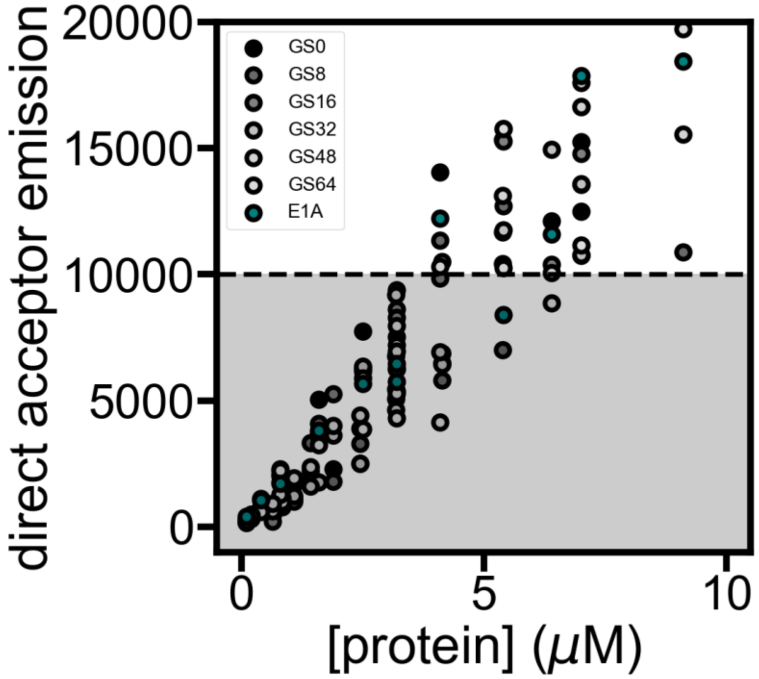

Figure S20. *In vitro* measurement of direct acceptor emission for known recombinant, purified proteins measured on the same setup as the live cells. Dashed line shows the emission cutoff used to select cells with a concentration range around 5 μM or lower to correlate with *in vitro* experiments.

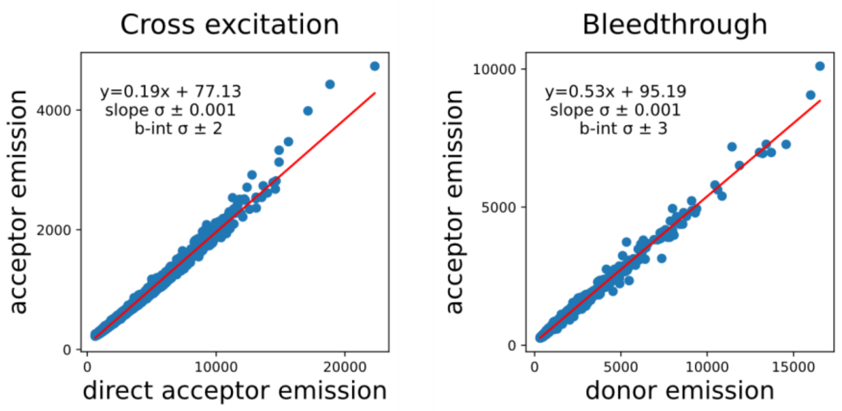

Figure S21. Measurements of cross-excitation (left) and bleedthrough (right) from donor to acceptor channel. To calculate cross-excitation, cells expressing mNeonGreen only were imaged. To calculate bleedthrough, cells expressing mTurquoise2 only were imaged. In both cases, the same imaging settings as those used for FRET constructs were used. (left) The x-axis shows acceptor emission under acceptor excitation. (right) The x-axis shows donor emission under donor excitation. In both figures, the y-axis shows acceptor emission under donor excitation. The slopes of these two values were used to correct the signal from the FRET construct according to the following equation:

$$F_A = F_{A,uncorrected} - (0.19 \times F_{A,direct} + 0.53 \times F_D)$$

where F_A is used to calculate E_f^{cell}. The numbers 0.19 ± 0.001 and 0.53 ± 0.001 are the slopes from the figures above.

Additionally, we performed photobleaching experiments where mNeonGreen of various FRET constructs were bleached. These bleached constructs were used to measure and calculate bleedthrough and similar results were obtained (slope of 0.51 ± 0.007).

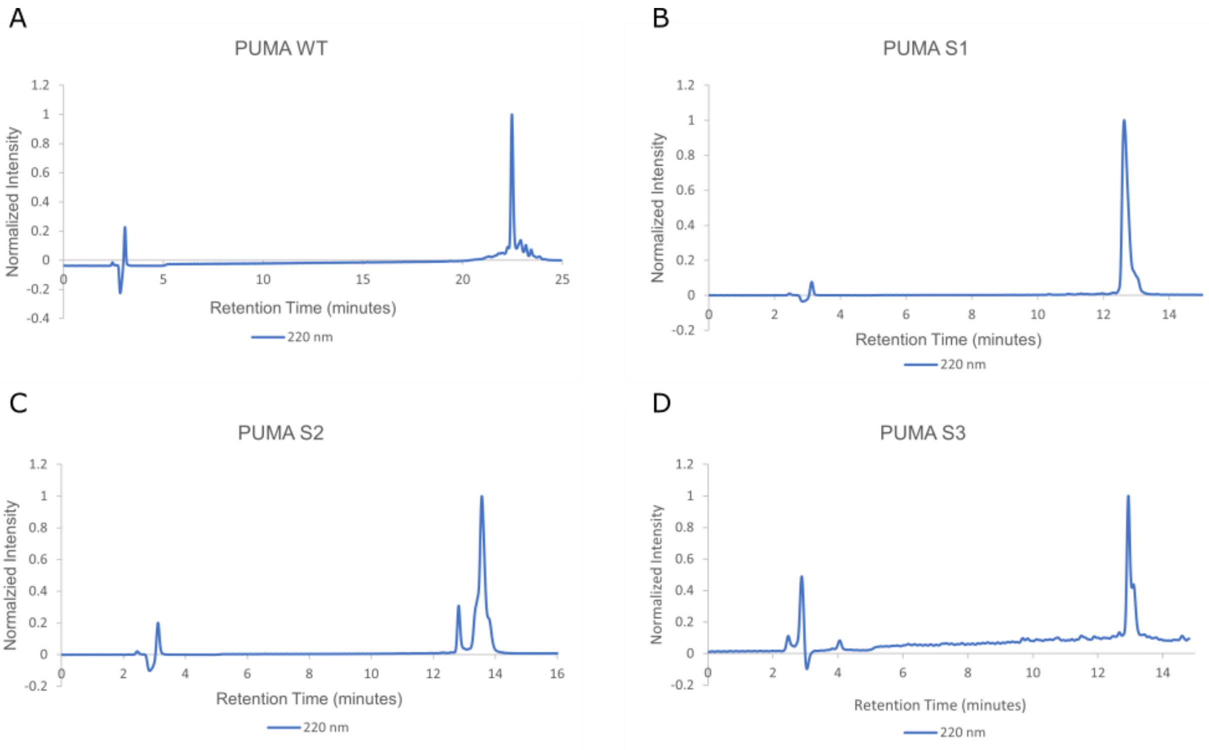

Figure S22. HPLC traces from purification of label-free peptides. **(A)** PUMA WT. **(B)** PUMA S1. **(C)** PUMA S2. **(D)** PUMA S3.

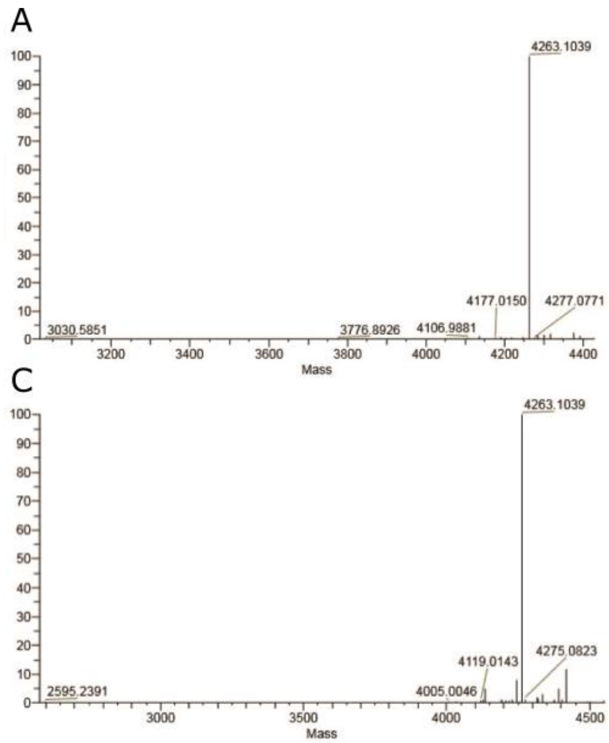

Figure S23. High-resolution ESI mass spectra of purified label-free peptides. (A) PUMA WT. (B) PUMA S1. (C) PUMA S2. (D) PUMA S3. Calculated and experimental masses are shown in **Table S5**.

Peptide	Calculated Mass (Da)	Experimental Mass from ESI-MS (Da)	Retention Time (minutes)
PUMA WT	4263.0918	4263.0974	22.47
PUMA S1	4263.0918	4263.1014	12.64
PUMA S2	4263.0918	4263.0923	13.59
PUMA S3	4263.0918	4263.0948	12.94

Table S5. Calculated and experimental masses of label-free peptides.

References

1. Rogers, J. M., Wong, C. T. & Clarke, J. Coupled Folding and Binding of the Disordered Protein PUMA Does Not Require Particular Residual Structure. *J. Am. Chem. Soc.* **136**, 5197–5200 (2014).
2. Vitalis, A. & Pappu, R. V. ABSINTH: a new continuum solvation model for simulations of polypeptides in aqueous solutions. *J. Comput. Chem.* **30**, 673–699 (2009).
3. Holehouse, A. S., Das, R. K., Ahad, J. N., Richardson, M. O. G. & Pappu, R. V. CIDER: Resources to Analyze Sequence-Ensemble Relationships of Intrinsically Disordered Proteins. *Biophys. J.* **112**, 16–21 (2017).
4. Mastop, M. *et al.* Characterization of a spectrally diverse set of fluorescent proteins as FRET acceptors for mTurquoise2. *Sci. Rep.* **7**, 11999 (2017).
5. Shaner, N. C. *et al.* A bright monomeric green fluorescent protein derived from Branchiostoma lanceolatum. *Nat. Methods* **10**, 407–409 (2013).
6. Baker, N. A., Sept, D., Joseph, S., Holst, M. J. & McCammon, J. A. Electrostatics of nanosystems: application to microtubules and the ribosome. *Proc. Natl. Acad. Sci. U. S. A.* **98**, 10037–10041 (2001).
7. Sievers, F. *et al.* Fast, scalable generation of high-quality protein multiple sequence alignments using Clustal Omega. *Mol. Syst. Biol.* **7**, 539 (2011).
8. Moses, D. *et al.* Revealing the Hidden Sensitivity of Intrinsically Disordered Proteins to their Chemical Environment. *J. Phys. Chem. Lett.* **11**, 10131–10136 (2020).
9. Vancraenenbroeck, R., Harel, Y. S., Zheng, W. & Hofmann, H. Polymer effects modulate binding affinities in disordered proteins. *Proc. Natl. Acad. Sci. U. S. A.* (2019) doi:10.1073/pnas.1904997116.
10. Pegram, L. M. & Record, M. T., Jr. Thermodynamic origin of Hofmeister ion effects. *J. Phys. Chem. B* **112**, 9428–9436 (2008).
11. Hopkins, J. B., Gillilan, R. E. & Skou, S. BioXTAS RAW: a free open-source program for small-angle X-ray scattering data reduction. *Foundations of Crystallography* **74**, a219 (2018).
