# Supplementary material for: Structural biases in disordered proteins are prevalent in the cell
Source: Nat Struct Mol Biol. 2024 Jan 4;31(2):283–92. doi: 10.1038/s41594-023-01148-8 (PMC10873198; doi:10.1038/s41594-023-01148-8)
Supplement: Supplementary file 2 — Reporting Summary [file 41594_2023_1148_MOESM2_ESM.pdf]

nature portfolio

Corresponding author(s): Shahar Sukenik

Last updated by author(s): Aug 13, 2023

Reporting Summary

Nature Portfolio wishes to improve the reproducibility of the work that we publish. This form provides structure for consistency and transparency in reporting. For further information on Nature Portfolio policies, see our Editorial Policies and the Editorial Policy Checklist.

Statistics

For all statistical analyses, confirm that the following items are present in the figure legend, table legend, main text, or Methods section.

n/a	Confirmed	
☐	☒	The exact sample size (*n*) for each experimental group/condition, given as a discrete number and unit of measurement
☐	☒	A statement on whether measurements were taken from distinct samples or whether the same sample was measured repeatedly
☐	☒	The statistical test(s) used AND whether they are one- or two-sided<br>*Only common tests should be described solely by name; describe more complex techniques in the Methods section.*
☒	☐	A description of all covariates tested
☒	☐	A description of any assumptions or corrections, such as tests of normality and adjustment for multiple comparisons
☐	☒	A full description of the statistical parameters including central tendency (e.g. means) or other basic estimates (e.g. regression coefficient) AND variation (e.g. standard deviation) or associated estimates of uncertainty (e.g. confidence intervals)
☐	☒	For null hypothesis testing, the test statistic (e.g. *F*, *t*, *r*) with confidence intervals, effect sizes, degrees of freedom and *P* value noted<br>*Give P values as exact values whenever suitable.*
☒	☐	For Bayesian analysis, information on the choice of priors and Markov chain Monte Carlo settings
☒	☐	For hierarchical and complex designs, identification of the appropriate level for tests and full reporting of outcomes
☒	☐	Estimates of effect sizes (e.g. Cohen's *d*, Pearson's *r*), indicating how they were calculated

Our web collection on statistics for biologists contains articles on many of the points above.

Software and code

Policy information about availability of computer code

Data collection	in vitro FRET data collected using BMG MARS software v5.4R3. SEC and SAXS data collected on the APS beamline using in-house software. CD data collected using JASCO Spectra Manager v2.14.06. Imaging data collected using Zen Blue v2.6. All simulations performed using CAMPARI v2
Data analysis	Unless otherwise stated, all data was analyzed using custom python code available at https://github.com/sukeniklab/IDP_structural_bias. SAXS data analyzed using BioXTAS RAW v2.1. Microscopy images analyzed with ImageJ v1.53. Simulation analysis performed using MDTraj v1.9.4.

For manuscripts utilizing custom algorithms or software that are central to the research but not yet described in published literature, software must be made available to editors and reviewers. We strongly encourage code deposition in a community repository (e.g. GitHub). See the Nature Portfolio guidelines for submitting code & software for further information.

Data

Policy information about availability of data

All manuscripts must include a data availability statement. This statement should provide the following information, where applicable:

- Accession codes, unique identifiers, or web links for publicly available datasets
- A description of any restrictions on data availability
- For clinical datasets or third party data, please ensure that the statement adheres to our policy

All data needed to evaluate the conclusions in the paper are present in the paper and its supporting information, as well as on the accompanying github repository available at https://github.com/sukeniklab/IDP_structural_bias. All the plasmids used in this study are available from the corresponding authors upon reasonable request. Some figures make use of PDB structures with accession codes 4AR7 and 5LTR. Source data are provided with this paper.

Research involving human participants, their data, or biological material

Policy information about studies with human participants or human data. See also policy information about sex, gender (identity/presentation), and sexual orientation and race, ethnicity and racism.

Reporting on sex and gender	N/A
Reporting on race, ethnicity, or other socially relevant groupings	N/A
Population characteristics	N/A
Recruitment	N/A
Ethics oversight	N/A

Note that full information on the approval of the study protocol must also be provided in the manuscript.

Field-specific reporting

Please select the one below that is the best fit for your research. If you are not sure, read the appropriate sections before making your selection.

☒ Life sciences ☐ Behavioural & social sciences ☐ Ecological, evolutionary & environmental sciences

For a reference copy of the document with all sections, see nature.com/documents/nr-reporting-summary-flat.pdf

Life sciences study design

All studies must disclose on these points even when the disclosure is negative.

Sample size	Sample sizes are provided for all experiments where N>30 in Table S4. Otherwise, individual datapoints are shown on the plots. Sample sizes are obtained from the numbers of cells imaged, with some exclusions as detailed below and in the methods.
Data exclusions	Some cells for live-cell data are filtered out based on specific criteria - under or over-expressed cells, cells with abnormal morphology following segmentation, and cells that have lifted off the coverslip following osmotic perturbations. This constituted < 10% of the imaged cells.
Replication	All experiments were conducted in two repeats at least. Repeats were conducted on different days, from different stock samples, and often from different protein expression batches. Live cell data was conducted on different passages, in different imaging chambers, and with individual transfections. All attempts at replication were successful.
Randomization	Different constructs were plated and imaged in different orders and different positions on well plates to ensure time prior to measurement is not a factor affecting the results
Blinding	Analysis of all raw data is fully automated and no blinding was used for this study

Reporting for specific materials, systems and methods

We require information from authors about some types of materials, experimental systems and methods used in many studies. Here, indicate whether each material, system or method listed is relevant to your study. If you are not sure if a list item applies to your research, read the appropriate section before selecting a response.

Materials & experimental systems

n/a	Involved in the study
☒	☐ Antibodies
☐	☒ Eukaryotic cell lines
☒	☐ Palaeontology and archaeology
☒	☐ Animals and other organisms
☒	☐ Clinical data
☒	☐ Dual use research of concern
☒	☐ Plants

Methods

n/a	Involved in the study
☒	☐ ChIP-seq
☒	☐ Flow cytometry
☒	☐ MRI-based neuroimaging

Eukaryotic cell lines

Policy information about cell lines and Sex and Gender in Research

Cell line source(s)	ATCC
Authentication	Cells were obtained directly from ATCC and no further authentication was performed
Mycoplasma contamination	Not tested for mycoplasma contamination
Commonly misidentified lines (See ICLAC register)	No commonly misidentified cells were used in this study
